# CShaperApp: Segmenting and analyzing cellular morphologies of the developing *Caenorhabditis elegans* embryo

**DOI:** 10.1002/qub2.47

**Published:** 2024-05-16

**Authors:** Jianfeng Cao, Lihan Hu, Guoye Guan, Zelin Li, Zhongying Zhao, Chao Tang, Hong Yan

**Affiliations:** ^1^ Department of Electrical Engineering City University of Hong Kong Hong Kong China; ^2^ Centre for Intelligent Multidimensional Data Analysis Limited Hong Kong China; ^3^ College of Computer and Information Hohai University Nanjing China; ^4^ Center for Quantitative Biology Peking University Beijing China; ^5^ Department of Biology Hong Kong Baptist University Hong Kong China; ^6^ State Key Laboratory of Environmental and Biological Analysis Hong Kong Baptist University Hong Kong China; ^7^ Peking‐Tsinghua Center for Life Sciences Peking University Beijing China; ^8^ School of Physics Peking University Beijing China; ^9^ Present address: Department of Computer Science University of Iowa Iowa City Iowa USA; ^10^ Present address: Dana‐Farber Cancer Institute and Department of Systems Biology Harvard Medical School Boston Massachusetts USA; ^11^ Present address: Department of Computer Science and Engineering Chinese University of Hong Kong Hong Kong China

**Keywords:** *C. elegans* embryogenesis, cellular morphology, cellular segmentation, deep learning, desktop software

## Abstract

*Caenorhabditis elegans* has been widely used as a model organism in developmental biology due to its invariant development. In this study, we developed a desktop software CShaperApp to segment fluorescence‐labeled images of cell membranes and analyze cellular morphologies interactively during *C. elegans* embryogenesis. Based on the previously proposed framework CShaper, CShaperApp empowers biologists to automatically and efficiently extract quantitative cellular morphological data with either an existing deep learning model or a fine‐tuned one adapted to their in‐house dataset. Experimental results show that it takes about 30 min to process a three‐dimensional time‐lapse (4D) dataset, which consists of 150 image stacks at a ∼1.5‐min interval and covers *C. elegans* embryogenesis from the 4‐cell to 350‐cell stages. The robustness of CShaperApp is also validated with the datasets from different laboratories. Furthermore, modularized implementation increases the flexibility in multi‐task applications and promotes its flexibility for future enhancements. As cell morphology over development has emerged as a focus of interest in developmental biology, CShaperApp is anticipated to pave the way for those studies by accelerating the high‐throughput generation of systems‐level quantitative data collection. The software can be freely downloaded from the website of Github (cao13jf/CShaperApp) and is executable on Windows, macOS, and Linux operating systems.

## 1 INTRODUCTION

Since *Caenorhabditis elegans* was adopted as a model organism, it has become a popular subject for numerous studies in developmental biology [1]. The inherent advantages of *C. elegans*, including its transparent body, small size, short lifespan, and highly invariant developmental pattern regarding cell position and cell lineage, significantly contribute to its popularity as a research model. From the fertilized egg to the end of embryogenesis, with approximately 550 living cells, each cell of *C. elegans* can be unambiguously named and tracked [2]. Importantly, its embryogenesis can be visually inspected in vivo using advanced fluorescence microscopy, which captures different cellular structures such as the cell nucleus and cell membrane, with specific fluorescence markers [3]. However, the image quality of microscopy suffers from physical limitations, such as photobleaching and phototoxicity. Thus, the large‐scale imaging data with a relatively low signal‐to‐noise ratio imposes serious challenges in systematic documentation and investigation of the developmental process.

Remarkably, the emergence of computer‐assisted methods has revolutionized the way to explore cellular information in *C. elegans* embryogenesis. Well‐established tools, such as StarryNite [4] and AceTree [5], have been developed to recognize, track, and visualize the cell nuclei in a *C. elegans* embryo. However, they are insufficient for characterizing cellular morphological features, such as cell shape, cell volume, cell surface area, and cell–cell contact area, which demand clear identification of cell boundaries [6]. Although several previous works proposed customized algorithms to segment *C. elegans* embryo images at the single‐cell level [7, 8], user‐friendly software for morphological analysis is still urgently needed, especially for handling the developmental stage with hundreds of cells.

To fill the gap mentioned above, we previously proposed an automatic cellular segmentation framework CShaper [9], to extract cellular morphological features up to the 350‐cell stage in *C. elegans* embryogenesis. The delivered dataset has received increasing attention in developmental biology research [10, 11, 12]. To improve the usability of CShaper to this community, in this work, we developed desktop software CShaperApp that interactively performs image segmentation and morphological analysis. Modular functions of CShaperApp allow researchers to either adaptively retrain CShaper with their own dataset or process external datasets with our pretrained model. The codebase is publicly available and can be easily modified for other animal organisms.

## 2 RESULTS

### 2.1 Software description

**FIGURE 1:**
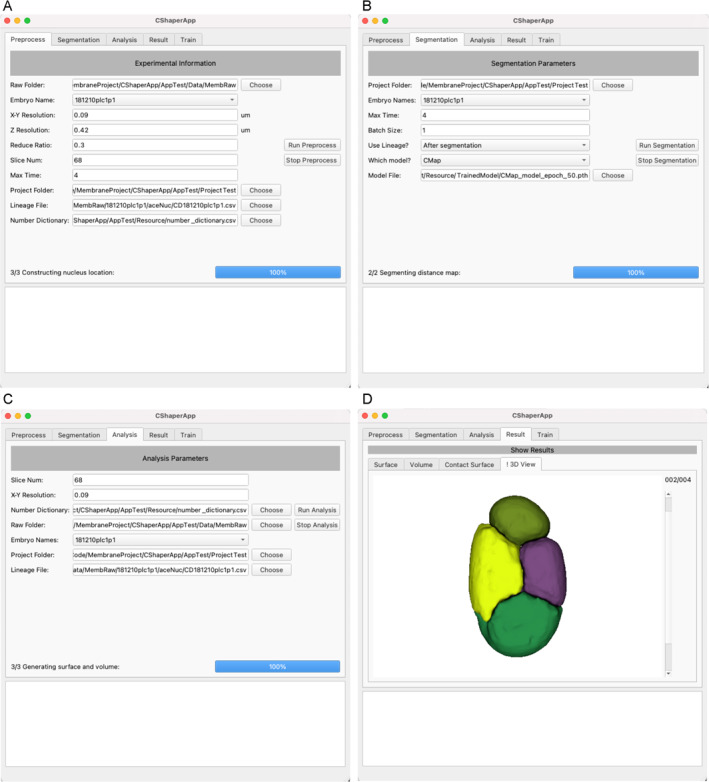
CShaperApp consists of five functional components: (A) *Preprocess*, (B) *Segmentation*, (C) *Analysis*, (D) *Result*, and *Train* for model retraining adapted to different datasets.

CShaperApp was developed with TensorFlow and PyQt5. To increase the flexibility of CShaperApp, the workflow was decomposed into a series of modules. Each module can be executed independently according to the requirements of different tasks. Specifically, CShaperApp consists of five modules, *Preprocess*, *Segmentation*, *Analysis*, *Result*, and *Train*. The *Preprocess* module stacks all microscopic slices at each time point into one volumetric image (Figure 1A). When the cell position and lineage file, generated from StarryNite and AceTree [4, 5], is available, the cell (nucleus) position information is extracted to synthesize binary nucleus masks where foreground pixels correspond to the centers of the cell nucleus. The *Segmentation* module adopts the CShaper framework to segment fluorescence images of cell membranes, transforming them into digitized segmentations with clear identification of the region corresponding to each cell (Figure 1B). The *Analysis* module automatically collects cellular morphological features such as cell volume, cell surface area, and cell–cell contact area, in the format of Excel (Figure 1C). The *Result* module provides the visualization of segmented cellular morphologies while showing all statistical information as well. Particularly, the segmented 3D embryo is shown with each cell labeled in different colors (Figure 1D). Users can apply quality control to statistical data to filter out confident results. The *Train* module allows users to retrain the model based on a specific dataset to deliver optimal results.

The modular pipeline allows users to work on the data flexibly. For example, the *Preprocess* and *Segmentation* modules are still executable even when the cell position and lineage file are unavailable, alleviating hands‐on time as users may be only interested in the overall cellular morphological changes regardless of cell identity. The integration of *Train* module enables researchers to optimize models using their own data in new application scenarios. CShaperApp can be executed on Windows, Linux, and MacOS, and all parameters used in different modules are configurable through user interfaces. Exemplary datasets, along with detailed usage guidelines, are available online for users to test and improve the performance of CShaperApp.

### 2.2 Numerical experiments

Computational efficiency and segmentation accuracy are two of the most important aspects in evaluating the performance of cellular segmentation software. We evaluated the computational efficiency with the time‐lapse images published in Ref. [9]. For segmentation accuracy, we applied CShaperApp to the datasets from Ref. [7, 8], which were captured from different equipment at various spatial resolutions. These numerical experiments could faithfully demonstrate the robustness of the program, thereby ensuring its reliability in a wide range of practical application scenarios.

The tested sample from the CShaper dataset includes 150 time points (covering the 4‐cell to 350‐cell stages) with a temporal resolution of 1.39 min [9]; the axial (*z*) resolution is 0.09 μm, and the lateral resolution (*xy*) is 0.42 μm. To segment these time‐lapse images, the *Preprocess* module first stacks all 68 slices at each time point together; then the volumetric image is resized from 512 × 712 × 68 to 153 × 213 × 95 with an isotropic spatial resolution of 0.30 μm. The *Segmentation* and *Analysis* modules are subsequently applied to get the final cellular morphological features. For a workstation with Intel® Xeon CPU and NVIDIA GPU 3090, it costs about 30 min in total to finish all the tasks. We recommend that users refer to the online document for specific usage guidelines, in particular to the file structure and data format.

**FIGURE 2:**
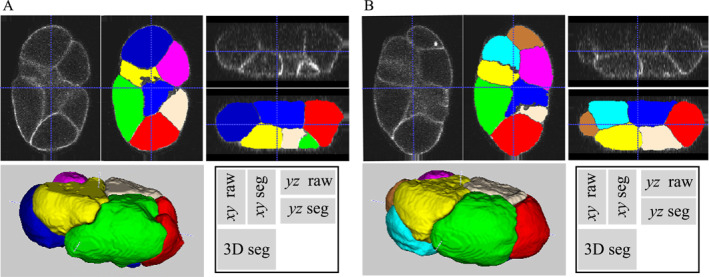
Experiment results on the dataset from spheresDT/Mpacts‐PiCS. Two cell segmentation examples of “TL2_2_t2‐6” processed with CShaperApp are shown in (A), (B), respectively. For the inset on the bottom right of both the left and right panels, “*xy*” and “*yz*” represent the direction of the observed cross‐sections, while “raw” and “seg” indicate the raw and segmented images, respectively.

Furthermore, we used data in the spheresDT/Mpacts‐PiCS dataset to verify whether CShaperApp is applicable to data from other laboratories [8]. The imaging data has a lower axial resolution than that used in CShaper (1.20 vs. 0.42 μm), making it challenging for the pretrained model to recognize cell regions correctly. We therefore utilized the *Train* module to retrain the model with eight pairs of images and annotations, helping the deep learning model adapt to the specific data distributions. The retrained model delivered promising results as shown in Figure 2. To sum up, the experimental result demonstrates the adaptability of CShaperApp to lower‐resolution images.

**FIGURE 3:**
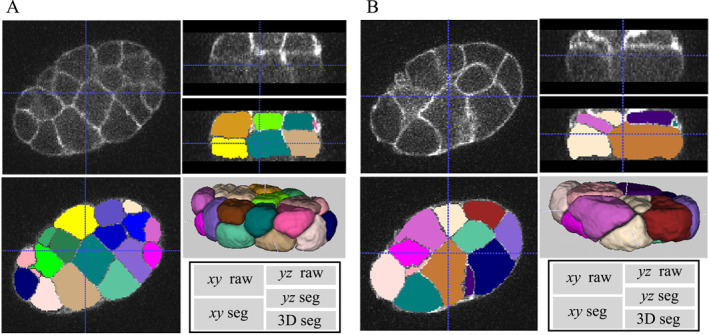
Experiment results on the dataset from BCOMS. Two cell segmentation examples at the 12‐ and 24‐cell stages processed with CShaperApp are shown in (A), (B), respectively. For the inset on the bottom right of both the left and right panels, “*xy*” and “*yz*” represent the direction of the observed cross‐sections, while “raw” and “seg” indicate the raw and segmented images, respectively.

Although fine‐tuning the deep learning model is favorable, researchers may face the problem of lacking annotated data for training. To investigate the scalability of CShaperApp, we utilized the previously trained model to process the data from BCOMS directly [7]. The data from BCOMS is imaged at almost isotropic spatial resolutions (axial resolution: 0.50 μm; lateral resolution: 0.44 μm) but its lateral resolution is lower than those of both CShaper and spheresDT/Mpacts‐PiCS. Without any bells and whistles, CShaperApp still preserves high performance on this dataset (Figure 3). The scalability of CShaperApp allows the users to leverage off‐the‐shelf tools in customized workflow.

CShaper treats a cluster of local minimum as seed in watershed segmentation, allowing for the detection of cavities among cells. However, it becomes challenging for CShaper to correctly recognize cells when the embryo develops into late stage. To demonstrate the flexibility of CShaperApp, we integrated CMap [13] as one of the optional models for specific purposes. Users may accordingly switch the segmentation model from CShaper into CMap so that low‐quality image can be better segmented by incorporating nucleus information. Specifically, as demonstrated in Ref. [13], there is no significant difference between CShaper and CMap before the 100‐cell stage; however, CShaper retains the superiority in detecting cavity. Therefore CShaper is particularly suitable for segmenting images before the 100‐cell stage in this integrated system. Additionally, modular design enables developers to reuse other components and reduce development costs.

## 3 DISCUSSION

CShaperApp is an efficient and scalable computer‐based application for researchers in developmental biology, facilitating the exploration of fine‐gained quantitative cellular morphological data in a developing embryo. Promisingly, such an integration of CShaper simplifies routine tasks in processing large‐amount microscopic images. Owning to the flexibility of modularized implementation, this open‐source tool can be easily optimized by replacing the integrated segmentation model with the more advanced ones.

## AUTHOR CONTRIBUTIONS

**Jianfeng Cao**: Conceptualization; investigation; validation; writing – original draft; writing – review & editing. **Lihan Hu**: Investigation; validation; writing – original draft. **Guoye Guan**: Conceptualization; validation; project administration; writing – original draft; writing – review & editing. **Zelin Li**: Validation. **Zhongying Zhao**: Funding acquisition; supervision; resources; writing – review & editing. **Chao Tang**: Funding acquisition; supervision; resources; writing – review & editing. **Hong Yan**: Funding acquisition; supervision; resources; writing – review & editing.

## CONFLICT OF INTEREST STATEMENT

The authors Jianfeng Cao, Lihan Hu, Guoye Guan, Zelin Li, Zhongying Zhao, Chao Tang, and Hong Yan declare no competing interests.

## ETHICS STATEMENT

This article does not contain any studies involving animals or human participants performed by any of the authors.

## Data Availability

The CShaperApp software and computation presented in this work can be found on the website of GitHub (cao13jf/CShaperApp).
